# Effects of Pore–Crack Relative Location on Crack Propagation in Porous Granite Based on the Phase-Field Regularized Cohesion Model

**DOI:** 10.3390/ma16237474

**Published:** 2023-12-01

**Authors:** Shiyi Zhang, Qiang Shen

**Affiliations:** School of Applied Science, Taiyuan University of Science and Technology, Taiyuan 030024, China; 15535111739@163.com

**Keywords:** phase-field regularization cohesion model, porous granite numerical simulation, deflection angle, damage behavior

## Abstract

This study employs the phase-field regularized cohesion model (PF-CZM) to simulate crack propagation and damage behavior in porous granite. The impact of the pore radius (r), initial crack–pore distance (D), and pore–crack angle (θ) on crack propagation is investigated. The simulation findings reveal that, with a fixed deflection angle and initial crack–pore distance, larger pores are more likely to induce crack extension under identical loading conditions. Moreover, with r and θ remaining constant, the crack extension can be divided into two stages: from its initiation to the lower edge of the pore and then from the lower edge to the upper boundary of the model. Multiple combinations of different D/r ratios and pore radii are derived by varying the values of D and r. These results demonstrate that with a constant r, cracks tend to deflect towards the pore closer to the initial crack. Conversely, when D remains constant, cracks will preferentially deflect toward pores with a larger r. In summary, the numerical simulation of rock pores and initial cracks, based on the PF-CZM, exhibits remarkable predictive capabilities and holds significant potential in advancing rock fracture analyses.

## 1. Introduction

The pore structure within rock masses encompasses heterogeneous elements such as voids, pores, and cracks [1]. Numerous studies have revealed that the presence of pores in rocks triggers stress redistribution in their proximity, which may induce brittle fractures at the pores and increase the permeability of the surrounding rock [2,3]. Therefore, it becomes imperative to investigate the initiation and progression of stress-induced microcracks in rocks. Rocks serve as a natural building material in various aspects of our lives, from structures founded on rock bases to concrete gravity dams, tunnels, and more [4]. These rock masses exhibit a porous matrix with embedded discontinuities, including bedding planes, faults, joints, and artificial cracks [5]. Among these discontinuities, cracks are the most prevalent form of defects, with a variety of applications spanning blasting, rock permafrost [6], hydraulic fracturing [5], and slope stability analysis. The uncontrolled proliferation of cracks within rock formations can result in structural damage, culminating in landslides, tunnel collapses, etc. Predicting crack growth has always constituted a paramount research challenge. A comprehensive understanding of the behavior of cracked materials is pivotal for evaluating their overall reliability.

Numerous experiments and numerical simulations have been deployed to scrutinize the intricate behavior of rocks containing defects, encompassing aspects such as crack initiation, propagation, interconnection, and linkage. The numerical manifold method was proposed to analyze the initiation and propagation of frictional cracks in rocks [7]. The failure mechanism of non-persistent joints in mortar subjected to compression was studied based on combining the extended finite element method (XFEM) with experiments [8]. The deformation and failure of rock materials and the diffusion of the flow and temperature within the context of multi-physical fields were simulated with a thermos–hydro–mechanical coupling peridynamic (PD) model [9]. The fracture process of rock samples already bearing flaws under uniaxial compression was simulated based on an improved smoothed particle hydrodynamics method (ISPH) [10]. The mechanisms governing the crack extension in both single- and double-cracked rocks under tensile and shear stress conditions were investigated with a combination of experimental observation and numerical simulation [11]. The mixed-mode fractures in rock-like materials were studied based on an extended peridynamic model equipped with a novel bond-breakage criterion [12]. The chemical effects on rock fracturing were explored with the extended finite element method (XFEM) [13].

Recently, the phase-field model (PFM) [14,15,16] has emerged as a highly promising tool for fracture mechanics calculations. What sets it apart from other models is its capacity to determine crack initiation, propagation, branching, merging, and other processes without the need for ad hoc failure criteria [17]. Duan et al. [18] employed a dynamic phase-field model to anticipate the rock fracture diversity under impact loading. Liu et al. [19] introduced a thermodynamically consistent phase-field model to simulate mixed-mode fractures in rock-like materials. Li et al. [20] applied a hybrid phase-field method for modeling to investigate mixed-mode fractures in elastoplastic rock-like materials. Meanwhile, Xu et al. [21] developed a new phase-field model, derived from the triple-shear energy criterion, to address mixed-mode brittle fractures in rocks. Moreover, PFM found extensive utility in a range of fracture scenarios, encompassing cohesive fractures [22,23,24], ductile fractures [25], brittle fractures [17,26,27], dynamic fractures [28,29], multi-physics fractures [30,31], and hyperelastic fractures [32,33,34].

Wong et al. [35,36] demonstrated that the crack growth path was significantly influenced by rock discontinuities and the porous rock structure. However, the influence of rock defect characteristics such as shape, size, distance, and relative position on crack propagation remains understudied. When cracks propagate within porous rocks, the way in which the surrounding pores control the crack path controlled by the surrounding pores remains complex and uncharted territory. Questions arise regarding which pores the crack tends to deflect towards and which specific pore’s deflection exerts a critical influence amid the multitude of pores. To delve into the effect of these features on crack extension in rock, the phase-field regularization cohesion model (PF-CZM) was employed to model and analyze the crack path propagation. The effects of pore radius (r) and pore distance (D) alone on rock expansion have been investigated in many studies; however, the coupling effects between prefabricated cracks and pore radius (r), initial crack–pore distance (D), and pore–crack angle (θ) have rarely been considered in granite with a double-pore structure. This research simulated the process of granite crack extension considering the coupling effect between three parameters based on a phase-field regularization cohesion model.

In rock fracture research, studies on rock fractures are mainly focused on single-pore media, and there is a lack of systematic research on the fracture behavior of porous media. In this work, a novel unified phase-field regularized cohesive force model was proposed to study the expansion of porous media in rock, especially for double-pore structures. This model allowed us to describe the fracture behavior in rocks in a continuous way, avoiding the discretization problem in the traditional methods and improving the numerical stability and convergence of the model. In this study, the accuracy of the model was validated through experimental verification and numerical simulation. Two classical rock fracture cases were simulated and analyzed to demonstrate the effectiveness and accuracy of the phase-field regularized cohesive model in predicting rock fracture behavior.

## 2. Materials and Methods

**Figure 1:**
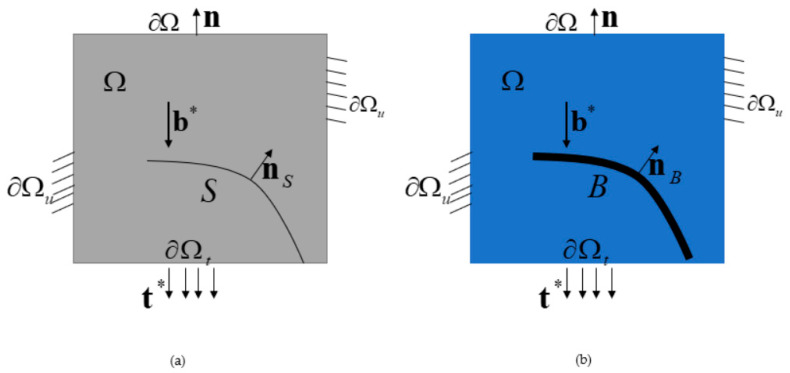
(**a**) A solid domain with sharp cracks; (**b**) geometric regularization.

In this section, we provide a concise overview of the phase-field theory of damage and fracture [17]. Figure 1a illustrates the solid domain $\Omega \subset {\mathbb{R}}^{n_{\dim}}\left({}^{n_{\dim}}=1,2,3\right)$ of the embedded crack. Within this context, we denoted the external boundary as $\partial \Omega ={\mathbb{R}}^{{}^{n_{\dim}}-1}$ and the outward normal vector as **n**. The deformation of the solid primarily results from both body forces $b^{*}$ distributed in the cracking solid Ω and tractions $t^{*}\left(x\right)$ applied to the boundary $\partial \Omega_{t}\subset \partial \Omega$. This deformation state is characterized by the displacement field u(x) and infinitesimal strain field $\varepsilon \left(x\right):=\nabla^{s}u\left(x\right)$, where x represents the spatial coordinate system and $\nabla^{s}\left(\cdot \right)$ is the symmetric gradient operator. For a well-posed boundary value problem, the external boundary ∂Ω is divided into two complementary segments, denoted as $\partial \Omega_{u}$ and $\partial \Omega_{t}$. We then applied a given displacement boundary $u^{*}\left(x\right)$ and force boundary $t^{*}\left(x\right)$ to their respective segments.

The sharp crack S was dispersed into a crack band B⊂Ω of a finite scale b>0, and a crack-phase field d(x):B⇒[0,1] was introduced to describe the crack state. The crack phase field adheres to the irreversibility condition $\dot{d}\left(x\right)\geq 0$, which satisfies d(x)=0 for elastic domains and d(x)=1 for cracks. It is imperative to underscore that the crack band B is not predetermined or held fixed throughout solid damage destruction but is automatically updated during crack propagation.

### 2.1. Governing Equations

The phase-field fracture theory is underpinned by the coupled damage–displacement problem [37]. These governing equations are derived from the energy minimization principle [38,39]:

(1) $$\left\{ \begin{array}{ll} \nabla \cdot \sigma +b^{*}=0 & \;\mathrm{in}\;\Omega \\ \sigma \cdot n=t^{*} & \;\mathrm{on}\;\partial \Omega_{t} \end{array}\right.$$

(2) $$\left\{ \begin{array}{ll} \nabla \cdot q+Q\left(d\right)\leq 0 & \;\mathrm{in}\;\beta \\ q\cdot n_{\beta }\geq 0 & \;\mathrm{on}\;\partial \beta \end{array}\right.$$

where the damage flux q is conjugated to the damage gradient ∇d, and the divergence is balanced by the damage source Q(d):

(3) $$q=\frac{2b}{c_{\alpha }}G_{f}\nabla d,\;Q(d)=-\omega^{\prime }\left(d\right)\bar{Y}-\frac{1}{c_{\alpha }b}G_{f}\alpha^{\prime }\left(d\right)$$

where the finite length scale b>0 represents the width of the crack band. When b→0, it corresponds to a sharp crack. We introduced the scaling constant $c_{\alpha }=4\int_{0}^{1}\sqrt{\alpha \left(\beta \right)}d\beta$ to reproduce the fracture energy $G_{f}$ during full failure. These governing equations are characterized by a monotonically increasing crack geometry function α(d)∈[0,1] and a monotonic decreasing energy degradation function ω(d)∈[0,1], both of which fulfill the following conditions:

(4) $$\begin{array}{l} \alpha \left(0\right)=0,\alpha \left(1\right)=1,\alpha^{\prime }\left(d\right)\geq 0\\ \omega \left(0\right)=1,\omega \left(1\right)=0,\omega^{\prime }\left(d\right)\leq 0,\omega^{\prime }\left(1\right)=0 \end{array}$$

The term $\omega^{\prime }\left(1\right)=0$ is introduced to mitigate the issue of spurious damage expansion observed in the gradient-enhanced damage model [40,41].

### 2.2. Constitutive Theory

This paper focuses solely on the cracking behavior under monotonic loading conditions, with the simplest constitutive relations [37] shown as follows:

(5) $$\sigma =\omega \left(d\right)\bar{\sigma },\bar{Y}=\frac{1}{2E_{0}}{\langle {\bar{\sigma }}_{1}\rangle }^{2}$$

where $\bar{\sigma }$ represents the effective stress tensor and can be expressed as:

(6) $$\bar{\sigma }=C_{0}:\varepsilon ,C_{0}=\frac{E_{0}}{1+\upsilon_{0}}\left(I+{\bar{\upsilon }}_{0}1⊗1\right)$$

where $C_{0}$ is Young’s modulus; ${\bar{\sigma }}_{1}$ denotes the principal value of the effective stress tensor $\bar{\sigma }$; and $\overset{-}{\upsilon_{0}}$ is connected to Poisson’s ratio $\upsilon_{0}$ and depends on the stress state, i.e., in the case of uniaxial stress, ${\bar{\upsilon }}_{0}=\upsilon_{0}$, ${\bar{\upsilon }}_{0}=\upsilon_{0}/\left(1-\upsilon_{0}\right)$ for plane stress; in other cases, ${\bar{\upsilon }}_{0}=\upsilon_{0}/\left(1-2\upsilon_{0}\right)$.

### 2.3. Optimal Characteristic Functions

To maintain generality, the general expression satisfying condition (4) is as follows [37]:

(7) $$\left\{ \begin{array}{ll} \alpha \left(d\right)=\xi d+\left(1-\xi \right)d^{2} & \xi \in \left[0,2\right]\;\\ \omega \left(d\right)=\frac{{\left(1-d\right)}^{p}}{{\left(1-d\right)}^{p}+a_{1}d\cdot P\left(d\right)} & P\left(d\right)=1+a_{2}d+a_{3}d^{2} \end{array}\right.$$

With the parameters $p\geq 2,a_{1}>0$, $a_{2}$ and $a_{3}$ need to be determined. To ensure that the resulting crack geometry function α(d) strictly falls within the range of [0,1], we set the parameter ξ∈[0,2] . It is worth noting that numerous studies [42] have employed similar energy degradation functions.

### 2.4. Phase-Field Models for Brittle Fractures

Several popular brittle fracture phase-field models [38,43,44] are present as follows:

(8) $$\left\{ \begin{array}{l} AT2:\xi =0\Rightarrow \alpha \left(d\right)=d^{2},{\;c}_{\alpha }=2\\ AT1:\xi =1\Rightarrow \alpha \left(d\right)=d,{\;c}_{\alpha }=\frac{8}{3}\\ WN:\xi =2\Rightarrow \alpha \left(d\right)=2d-d^{2},{\;c}_{\alpha }=\pi \end{array}\right.$$

(9) $$p=2,a_{1}=2,a_{2}=-\frac{1}{2},a_{3}=0\Rightarrow \omega \left(d\right)={\left(1-d\right)}^{2}$$

For the above brittle fracture phase-field models, the calculation equation for the critical (peak) stress $\sigma_{c}$ is given in [27,44]:

(10) $$\sigma_{c}\left\{ \begin{array}{ll} \sqrt{\frac{27}{256}\frac{E_{0}G_{f}}{b}} & \;\mathrm{AT}2\\ \sqrt{\frac{3}{8}\frac{E_{0}G_{f}}{b}} & \;\mathrm{AT}1\\ \sqrt{\frac{2}{\pi }\frac{E_{0}G_{f}}{b}} & \;\mathrm{WN} \end{array}\right.$$

The critical stress $\sigma_{c}$ is tied to the length parameter b. Consequently, the entire load–displacement curve proves to be exceedingly sensitive to the length scale parameter b [45]. To address these challenges, in the phase-field model of brittle fractures, a common strategy is often employed. This involves treating the length scale b as a material property rather than merely a numerical parameter [39]. A prevalent approach is to equate the critical stress with the failure strength $f_{t}$:

(11) $$\sigma_{c}=f_{t}\Rightarrow \;b=\left\{ \begin{array}{ll} \frac{27}{256}l_{ch} & \;\mathrm{AT}2\\ \frac{3}{8}l_{ch} & \;\mathrm{AT}1\\ \frac{2}{\pi }l_{ch} & \;\mathrm{WN} \end{array}\right.$$

In brittle fracture phase-field models, the internal length $l_{ch}=E_{0}G_{f}/f_{t}^{2}$ serves to determine the dimensions of the fracture process zone, and a smaller internal length signifies the material’s greater susceptibility to brittleness.

### 2.5. Phase-Field Regularized Cohesive Zone Model

Comparatively, for any parameter ξ∈(0, 2], the energy degradation function (7) of a rational type leads to a set of phase-field damage models, with the following non-vanishing failure strength [46]:

(12) $$\sigma_{c}=\sqrt{\frac{2\xi E_{0}G_{f}}{c_{\alpha }}}\frac{1}{a_{1}b}=f_{t}>0$$

(13) $$a_{1}b=\frac{2\xi }{c_{\alpha }}l_{ch}\Rightarrow a_{1}=\frac{2\xi }{c_{\alpha }}\frac{l_{ch}}{b}$$

In other words, parameter $a_{1}>0$ is no longer the constant $a_{1}=2$, as typically seen in the brittle fracture phase-field model. Instead, it becomes a function related to parameter b. This adjustment ensures that the failure $f_{t}$ remains constant. Thus, the predicted global load–deformation response is insensitive to the length scale [37,44,47]. Consequently, maintaining favorable Γ-convergence is achievable by minimizing the regularization length scale b. Notably, when this length scale approaches zero, i.e., b→0, we can get the resulting phase-field model converges to a mixed-mode PF-CZM with general softening laws. Wu et al.’s work [46] illustrates more details.

In this paper, we refer to Wu et al.’s research [37] for the suggestion of using the crack geometry function of ξ=2, i.e.,

(14) $$\alpha \left(d\right)=2d-d^{2}\Rightarrow c_{\alpha }=\pi ,\;\;\;a_{1}=\frac{4}{\pi }\cdot \frac{l_{ch}}{b}$$

(15) $$a_{2}=2\beta_{k}^{2/3}-\left(p+\frac{1}{2}\right),a_{3}\left\{ \begin{array}{ll} 0 & \;p\;\text{>}\;2\\ \frac{1}{2}\beta_{\omega }^{2}-\left(1+a_{2}\right) & \;p=2 \end{array}\right.$$

The ratio of $\beta_{k}$ and $\beta_{\omega }$ depends on the initial slope $k_{0}$ and the limit crack opening $k_{0}$:

(16) $$\beta_{k}:=\frac{k_{0}}{-\frac{1}{2}f_{t}^{2}/G_{f}}\geq 1,\;\;\;\beta_{\omega }:=\frac{\omega_{c}}{2G_{f}/f_{t}}$$

Based on the linear softening curve, it can be deduced that $\beta_{k}=\beta_{\omega }=1$.

The resulting PF-CZM is considered optimal since it accommodates a wide range of softening laws commonly employed for brittle and quasi-brittle materials, including linear equations, exponential equations, hyperbolic equations, and the approach proposed by Sarac et al. [48]. Brittle fractures and the approach by Cornelissen et al. [49] are a popular choice for linear softening curves, aligning with Wu et al.’s findings [37]:

(17) $$\left\{ \begin{array}{lll} Linear\;\mathrm{softening}\;\mathrm{curve}; & \;p=2,\;a_{2}=-\frac{1}{2},\; & a_{3}=0\;\\ \mathrm{softening}\;\mathrm{curve}; & \;p=2,\;a_{2}=1.3868,\; & a_{3}=0 \end{array}\right.$$

The mentioned parameters $p,a_{1},a_{2}$ and $a_{3}$ are calibrated from the Type I failure mode. However, it is important to note that the PF-CZM can effectively handle mixed-mode failure, with corresponding details shown in [46].

For the PF-CZM, i.e., ξ∈(0,2], we can draw the following conclusions based on Equations (7) and (13):

(18) $$\alpha^{,}\left(0\right)=\xi ,\;\;\;\omega^{,}\left(0\right)=-a_{1}=-\frac{2\xi }{c_{\alpha }}\frac{l_{ch}}{b}$$

Once the crack nucleates, i.e., d=0, the evolution equation for the crack phase field becomes:

(19) $$Q_{0}=-\omega^{,}\left(0\right)\overset{-}{Y_{{}_{0}}}-\frac{1}{c_{\alpha }b}G_{f}\alpha^{,}\left(0\right)=\frac{2\xi }{c_{\alpha }}\frac{l_{ch}}{b}\overset{-}{Y_{{}_{0}}}-\frac{1}{c_{\alpha }b}G_{f}\xi =0$$

Or, equivalently,

(20) $$\overset{-}{Y_{{}_{0}}}=\frac{1}{2}\frac{G_{f}}{l_{ch}}=\frac{1}{2E_{0}}f_{t}^{2}\Rightarrow {\overset{-}{\sigma }}_{1}=f_{t}$$

Therefore, as the maximum principal stress ${\overset{-}{\sigma }}_{1}$ reaches the failure strength $f_{t}$, the crack nucleation commences, indicating that the PF-CZM harmoniously incorporates a strength-based crack nucleation criterion, an energy-based crack propagation criterion, and a variational crack path selector into a single framework.

## 3. Simulation of Rock Fractures and Experimental Verification

In this section, we employed the PF-CZM for numerical simulation and experimental verification on rocks. We conducted two sets of experiments, namely, the SCB monotonic fracture test [39] and the granite tensile fracture test [1], on the rocks. By comparing the experimental results with those from the numerical simulation, we substantiated the precision of the PF-CZM in predicting rock crack propagation.

### 3.1. SCB Monotonic Fracture Test

**Figure 2:**
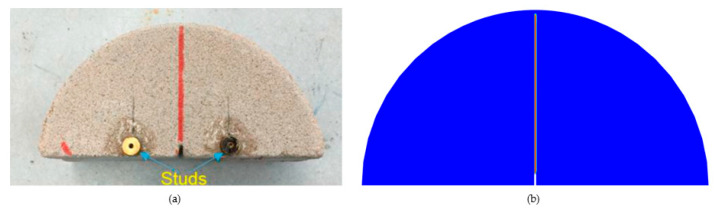
(**a**) Experimental results and (**b**) (PF-CZM) simulation results.

A typical cement-stabilized sand was characterized with the Semi-Circular Bend (SCB) fatigue test for crack propagation [39]. The sand was fine-grained quartz sand, in which the cement-stabilized sand cement content ranged from 3% to 11% of the weight of dry material. In the experiment, dry sand with 9% cement content was selected. The circular specimen was divided into two semi-circular specimens, and each semi-circular specimen was notched. The final semi-circular specimen had a thickness of 60 mm, a diameter of 150 mm, and a 5 mm notch. The semi-circular specimen with a pre-crack was placed in the MTS (Material Testing System) machine for monotonic SCB fracture testing. MTS was the testing equipment used for mechanical property testing of materials. Downward displacement was applied at a constant rate of 1.0 mm/min until the specimen fractured, repeating three times. Figure 2a illustrates the crack growth diagram under this test. In this research, COMSOL-Multiphysics 6.1 numerical simulation software was used to simulate and analyze. A phase-field regularized cohesive force model was applied to verify the experiment, ensuring that the geometry, boundary conditions, and loading rate matched those of the experimental setup. 

**Figure 3:**
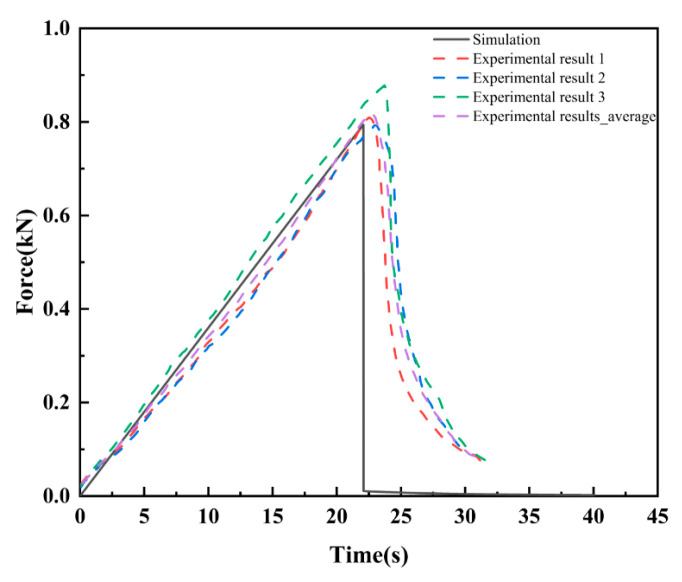
Load–displacement curves of the SCB disk.

Figure 2b depicts the crack propagation diagram of the SCB disk. Figure 3 depicts the load–displacement curves of the SCB disk. The crack in the SCB disk vertically extended upward to the top along the prefabricated crack direction. In addition, the experimental results described in this paper were supported by the simulation validation by Zhang et al. [39]. Many studies [50,51] have also conducted similar experiments, and the test results were very similar or consistent with the simulation results in this paper.

### 3.2. Granite Tensile Test

**Figure 4:**
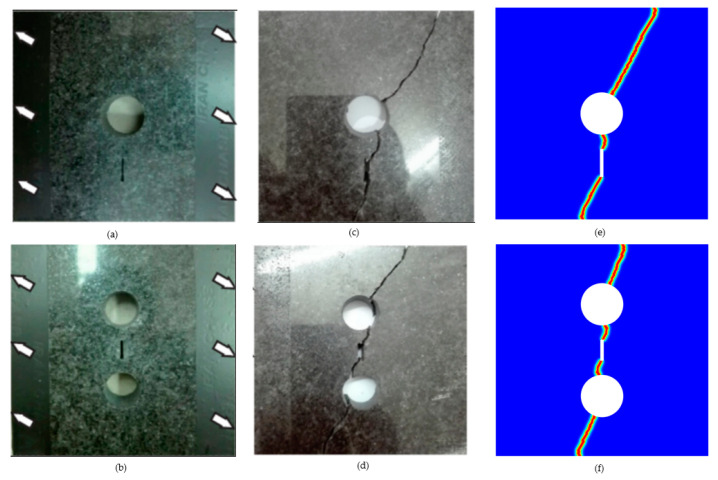
Two granite specimens: (**a**) one hole and one crack, (**b**) two holes and a crack, (**c**,**d**) experimental results, and (**e**,**f**) simulation results ((**a**–**d**) are the results from Rezanezhad et al.’s work [1]).

Rezanezhad et al. [1] investigated the crack propagation behavior of porous granite through an experimental analysis. Granite slabs of 80, 80, and 15 mm in length, width, and thickness were prepared, and the prefabricated crack and pores were cut with a water jet. The specimen was placed in an STM-250 tensile testing machine, which is a specialized device for conducting bi-axial tensile tests. The machine can apply tensile loads in multiple directions to simulate complex loading and mechanical behaviors under various conditions. Displacement loads were applied at a constant rate from both sides of the specimen at 30 degrees until the specimen fractured. The experimental crack path is depicted in Figure 4c,d. To replicate these experimental results, the PF-CZM was utilized, with the granite’s geometry, boundary conditions, and loading method aligning with the experimental setup, where the elastic modulus was E=70.6 GPa, Poisson‘s ratio was υ=0.25, and the fracture energy density was $G_{f}=38.5\;N/m$. The elastic modulus, Poisson’s ratio, and fracture energy density were from reference [1]. Figure 4e,f present the numerical results of the PF-CZM, demonstrating a remarkable agreement with the experimental results. In addition, the researcher also simulated the experiment based on the extended finite element method [1], and the results were very similar or consistent with the simulation results in this paper. Consequently, the PF-CZM proved to be highly dependable for simulating fractures in porous structures.

## 4. Results and Discussion

**Figure 5:**
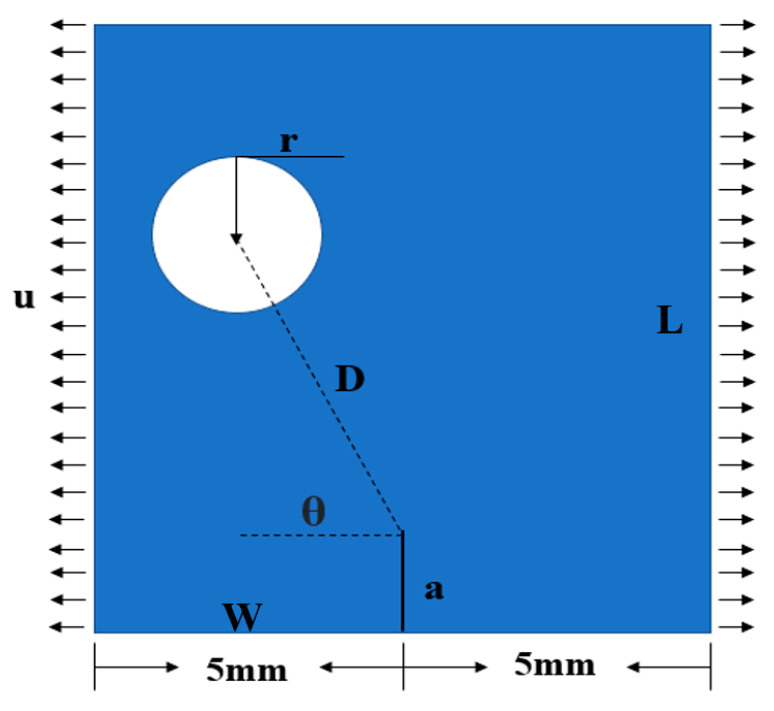
The relative location of pores and initial cracks.

In this section, we utilized the PF-CZM to numerically simulate the relative location of rock pores and initial cracks. Pores and cracks are typical defect patterns found in rocks. This model enabled us to analyze phenomena such as crack propagation and rock deformation in the rock, thus enhancing our understanding of the mechanisms governing rock failure by simulating the crack evolution under varying conditions. The relative location of pores and initial cracks are shown in Figure 5.

The initial crack length (a), rock slab length (L), and rock slab width (W) were 1, 15, and 10 mm, respectively. u is denoted as the displacement load. Our investigation primarily focused on three key aspects: (1) We explored the impact of varying pore sizes (r) on crack propagation. This variation allowed us to observe how pore size influenced rock fracture and strength. (2) Considering the variation of different pore-to-crack distances (D) on crack propagation, we gained insights into the varying D interaction effects on crack propagation by adjusting the value of D. (3) The pore–crack angle (θ) describes the deflection of the pore position relative to the crack tip. Varying the value of θ enabled us to assess the impact of the relative position of pores in the rock on the crack path propagation and velocity.

### 4.1. Effects of Pore Size (r)

To investigate the influence of varying r on crack propagation, we kept certain parameters constant: D = 4 mm, θ = 90°, and a = 1 mm. We explored different pore sizes, specifically r values of 0.2, 0.8, and 1.2 mm. A uniform displacement of 0.001 mm/s was applied to both the left and right sides of the model, simulating the crack propagation process, which allowed us to determine the influence of different pores on rock failure.

**Figure 6:**
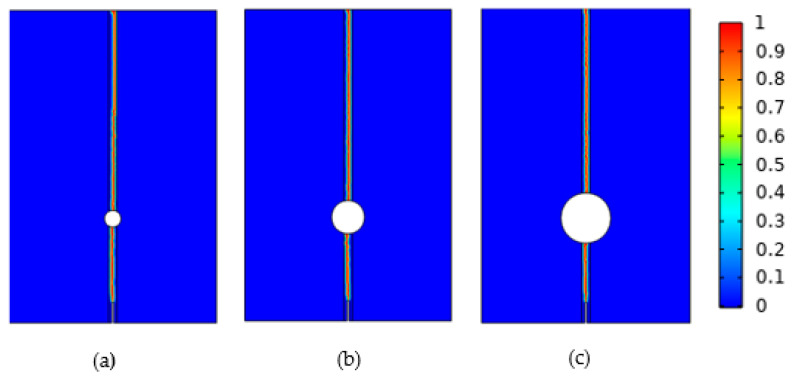
Crack propagation diagrams for different pore sizes: (**a**) 0.4 mm, (**b**) 0.8 mm, (**c**) 1.2 mm.

The fracture diagrams for crack propagation with different pore sizes are illustrated in Figure 6. The crack path is a straight line, originating from the upper end of the initial crack and extending upward through the pores to the highest point of the model. Furthermore, Zhang et al. [9] simulated the crack propagation of the rock based on the peridynamic model and the simulation results were consistent with the simulation results in this work, demonstrating the reliability of the phase-field regularized cohesion model.

**Figure 7:**
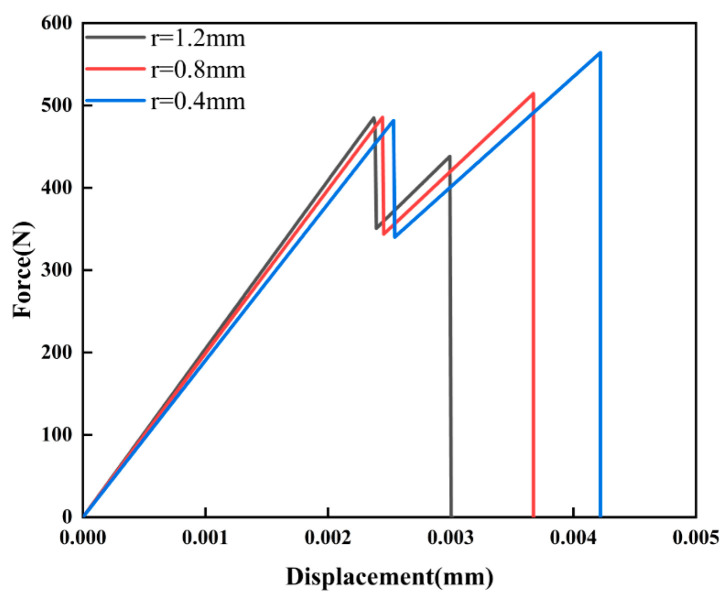
Load–displacement curves for different pore sizes.

From the load–displacement curves shown in Figure 7, it is evident that the pore radii of 0.4, 0.8, and 1.2 mm corresponded to the maximum reaction forces of 564, 514, and 484 N, respectively. Notably, as r increased, the maximum reaction force decreased by 8% and 14%, indicating that compared with smaller pore structures, larger pore structures were more prone to damage under the same loading conditions, When the uniform displacement was applied to both the left and right sides of the model, the larger pore structure was more prone to crack expansion, so only a small displacement was necessary to satisfy the requirement for crack propagation.

### 4.2. Effect of the Pore–Crack Distance (D)

In this section, we delved into the impact of the pore-to-crack distance (D) on crack propagation. Referring to the schematic diagram of the relative positions of pores and cracks in Figure 5, D represents the distance between the crack tip and the center of the pore circle. To facilitate our investigation, we kept other parameters constant and solely focused on varying the size of D. We established three models with fixed values: r = 0.4 mm, a = 1 mm, θ = 90°, and D = 1 mm, 1.5 mm, and 2 mm, respectively. These models were employed to simulate the crack propagation under a constant acceleration rate of 0.001 mm/s.

**Figure 8:**
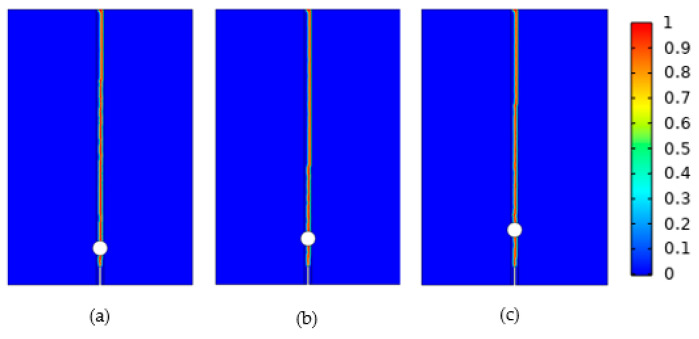
Crack propagation diagrams for different distances: (**a**) 1 mm, (**b**) 1.5 mm, (**c**) 2 mm.

Figure 8 provides a schematic representation of the crack propagation with varying Ds, which was similar to the r expansion, starting from the upper end of the initial crack and expanding vertically upwards throughout the pore until reaching the upper boundary of the model.

**Figure 9:**
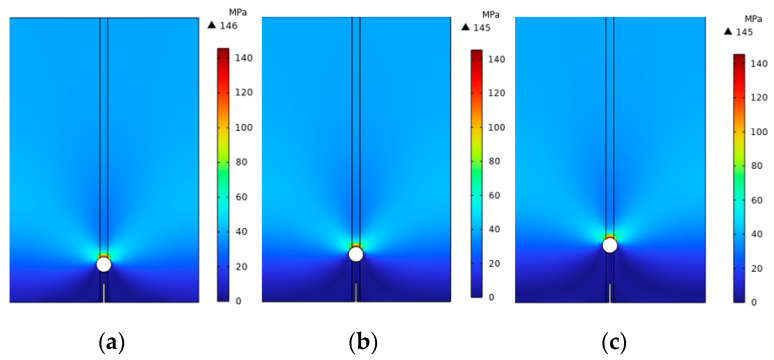
The von Mises stress distribution diagrams: (**a**) 1 mm, (**b**) 1.5 mm, (**c**) 2 mm.

**Figure 10:**
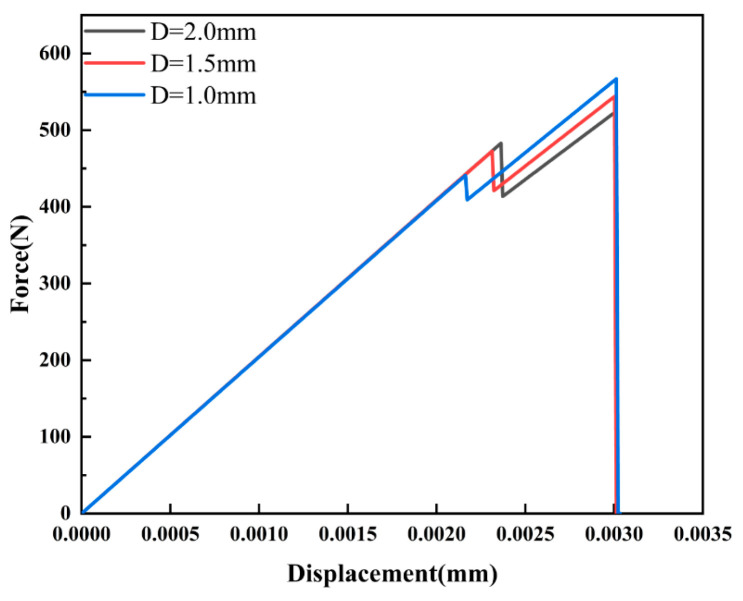
Load–displacement curves for different distances (Ds).

Figure 9 depicts the von Mises stress distribution at different distances. Interestingly, the von Mises stress distribution exhibited similarity across different pore radii. Especially at the crack tip, when the D was small, there was a pronounced stress concentration at the tip. However, as the D increased, the stress gradually dispersed and diminished, with stress levels in the tip region gradually decreasing as well. Figure 10 displays the load–displacement curves, which could be divided into two stages: first, crack initiation began to extend to the lower edge of the pore; second, the crack, having initiated at the lower edge of the pore, proceeded to extend upward until it reached the upper boundary of the model.

Throughout the process, from the crack initiation to its expansion towards the lower edge of the pore, the reaction force steadily increased. In this first stage, the maximum reaction force reached 440, 472, and 482 N for Ds of 1, 1.5, and 2 mm, respectively, and slightly rose by 7% and 9%. As the crack continued to expand from the lower edge to the upper edge, higher loads were applied compared with those of the previous stage. In the second stage, the maximum reaction force was 566, 543, and 522 N for Ds of 1, 1.5, and 2 mm, respectively, and slightly decreased by 4% and 7%. These results indicated that a higher load was required to resist crack propagation as the crack extended from the bottom edge to the top edge. This conclusion was consistent with Zhang et al.’s research [1] on similar problems.

### 4.3. Effect of Double Pores on Crack Extension

In the previous section, we explored various scenarios where the θ was 90°. In such cases, we observed that the typical pattern of crack growth involved a straight-line extension throughout the pore, reaching the upper edge. Meanwhile, the stress distribution in the crack tip region was usually symmetrical. However, when the angle between the pore and the crack was not 90°, crack propagation often deflected from a straight path toward the direction of pores at a specific angle, and the stress distribution in the crack tip region was no longer symmetrical. It should be noted that not all pores exhibit this effect, and only pores located at critical angles related to the crack tip can cause such crack deflection.

**Figure 11:**
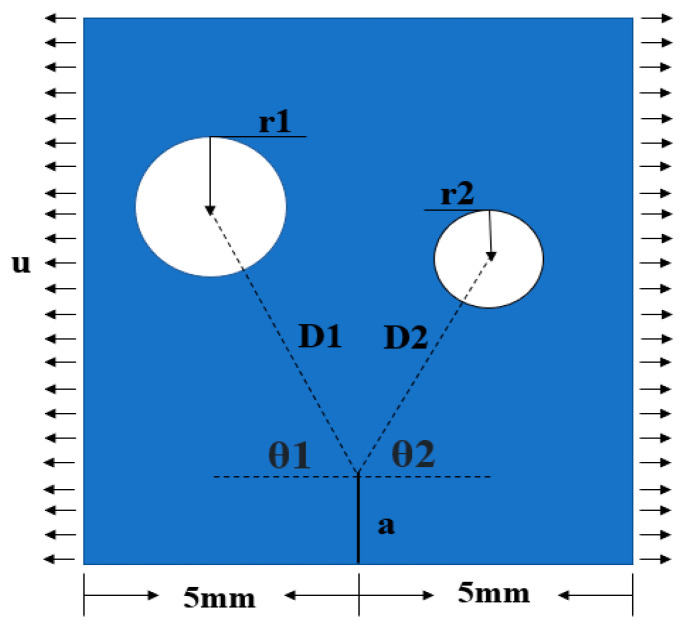
The relative position of double pores and the initial crack.

To visually illustrate the positional relationship between the angle θ of double pores and fractures, we created a simplified model, as depicted in Figure 11. We increased the angle θ to induce a deflection in the crack propagation path and studied how the propagation path of the crack deviated from its original straight path due to the influence of angle θ. To accomplish this, we developed three series of models, each comprising five models, as detailed in Table 1. The pore radius of each series was 0.4, 0.8, and 1.2 mm, respectively. By altering the ratio between D and r (D/r), we achieved a range of variations between two and six in each series.

Building upon the findings from Section 4.1 and Section 4.2, where we investigated the influence of r and D on crack extension, we now discuss the combined effect of r, D, and θ on crack deflection and extension. As shown in Figure 11, we constructed a model featuring double pores alongside an initial crack. These pores could induce crack deflection, but it was imperative to identify which of these pores played the decisive role in deflecting the cracks. According to Table 1, we simply divided the models into two types: those with different deflection angles and those with equal deflection angles.

#### 4.3.1. Effect of Different Deflection Angles on Crack Extension

**Figure 12:**
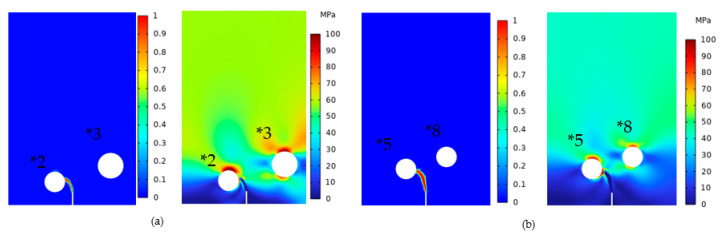
(**a**) Models *2 and *3 and (**b**) Models *5 and *8 crack propagation and von Mises stress.

To simulate the influence of different deflection angles on crack propagation, we employed four models, namely, Model *2, Model *3, Model *5, and Model *8, as listed in Table 1. This approach allowed us to accurately understand how each pore affected the path of crack growth. It is important to note that the length, position, and material properties of the initial crack remained constant. According to Table 1, the deflection angles of Models *2 and *3 were 30° and 35°, respectively. The crack propagation and von Mises stress distribution for these models are presented in Figure 12a. Evidently, the crack deflected towards the direction with a smaller deflection angle, and when the crack initiated deflection, the stress between the closer pores was significantly higher than that between the more distant pores. In regions with high stress, the crack exhibited a faster growth rate, while in areas with low stress, the crack growth rate was slower. Therefore, the crack tended to deflect towards the high-stress region, i.e., towards the closer pore, which was the preferred path for crack propagation.

Continuing the simulations for Models *5 and *8, which, as shown in Table 1, had deflection angles of 50° and 60°, respectively, we adhered to the established rule. Accordingly, the crack would deflect towards the closer pore. Figure 12b presents the crack propagation diagram and von Mises stress distribution cloud diagram for Models *5 and *8. Notably, the crack indeed deflected towards the closer pore, and the stress between the crack tip and the closer pore was significantly higher than that of the more distant pore, which conformed to the same rule observed in the previous simulation.

Based on the previous simulation results, we could draw the following conclusions. When r remained constant and D varied, the crack deflected towards the closer pore, indicating that D was the most influential parameter affecting crack deflection. Alterations in D gave rise to changes in stress distribution, which, in turn, affected the crack propagation rate between the regions of high and low stress and then determined the crack deflection direction.

#### 4.3.2. Effect of Equal Deflection Angles on Crack Extension

**Figure 13:**
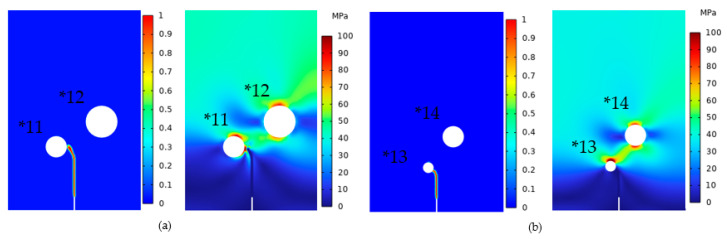
(**a**) Model *11 and *12 and (**b**) Model *13 and *14 crack extension and von Mises stress.

In Table 1, there are multiple models featuring the same deflection angle, which can be categorized into two groups. One group comprised models with an identical D/r ratio, while the other contained models with differing D/r ratios. We first investigated the models with similar D/r ratio such as Models *11 and *12 with a deflection angle of 70° and Models *13 and *14 with a deflection angle of 75°. This examination aimed to determine which pore exerted a more pronounced and influential role in deflecting the crack. As illustrated in Figure 13, the simulation results showed that the crack deflected towards the nearby pore, highlighting the pivotal role of the nearby pore in driving crack deflection. The figure also revealed the von Mises stress distribution between the crack tip and pore region. A high-stress region was evident between the crack tip and nearby pore, which was the primary factor contributing to crack deflection.

**Figure 14:**
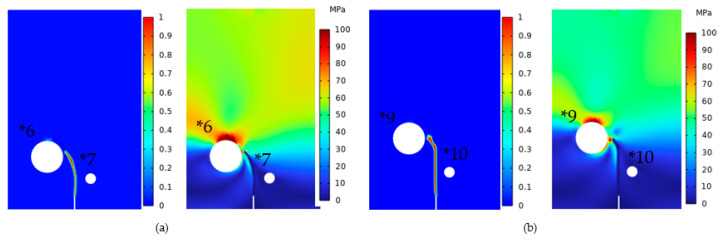
(**a**) Models *6 and *7 and (**b**) Models *9 and *10 crack extension and von Mises stress.

The second type of simulation adopted Models *6 and *7 (θ = 55°), in which the radii were 1.2 and 0.4 mm, respectively, and the ratios of D/r were three and four, respectively, and Models *9 and *10 (θ = 65°), in which the radii were 1.2 and 0.4 mm, respectively, and the ratios of D/r were three and four, respectively. With the crack propagation and von Mises stress distribution shown in Figure 14, it is obvious that the cracks would deflect towards the pores with smaller D/r ratios. These two types of simulations demonstrated that the deflection behavior of cracks exhibited a consistent law. Specifically, when the pores shared the same r, the crack would deflect towards the direction that had the smaller crack–pore distance (D). Conversely, when the pore had the same D, the crack tended to deflect towards the pores with a larger r. This rule could be simply described as follows: in such scenarios, the crack path inclined towards pores with smaller D/r ratios. This principle remained applicable across diverse instances, encompassing varying D/r ratios and pore radii, regardless of whether the θ was equal.

**Figure 15:**
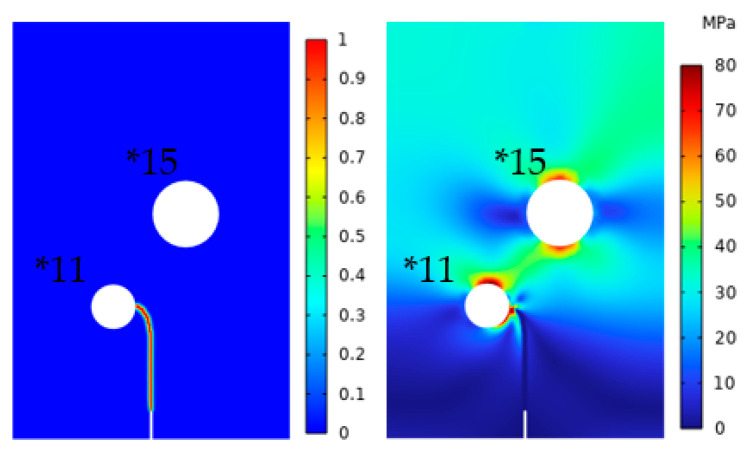
Models *11 and *15 crack extension and von Mises stress.

In order to verify the correctness of this principle, Models *11 and *15 were simulated in Table 1. The two models had radii of 0.8 and 1.2 mm, respectively, while the D/r ratios were five and six, respectively. The crack extension and von Mises stress distribution are shown in Figure 15. It was observed that the crack also deflected towards pores with smaller D/r ratios, which further verified the summarized rule.

## 5. Conclusions

This study employed the PF-CZM to verify the SCB disk fracture test, revealing a strong concurrence between our simulations and experimental results. Additionally, we applied this model to analyze porous granite and compared the simulation results with previously studied experimental results, which demonstrated an extremely high agreement between these results. This underscored the remarkable effectiveness of the PF-CZM as a numerical tool for precisely forecasting porous rock crack propagation. This study offered a dependable numerical tool, enhancing our comprehension of porous rock characteristics and damage across diverse conditions.

To investigate the effects of pores and initial cracks on crack propagation, three different simulations were carried out.

1. In a tensile model with an initial crack and two pores (with equal pore radii but different crack–pore distances (Ds)), the crack would deflect towards the pore with the smaller pore distance.
2. In a tensile model with an initial crack and two pores (with equal crack–pore distances (Ds) but different pore radii), the crack would deflect towards the pore with larger pore radii.
3. In a tensile model with an initial crack and two pores (with different crack–pore distances (Ds) and pore radii), the crack would deflect towards the pore with the smaller D/r ratio.

### Expectation

When rock materials are applied in complex environments such as deep seas and high temperatures, they will face challenges such as chemical corrosion and thermal strain, which can cause irreversible damage to rock structures. In this context, it is necessary to consider the effects of mechanical–chemical coupling and thermos–mechanical coupling. By establishing the mathematical model of mechanical, chemical, and temperature coupling, the crack propagation behavior of rock materials can be accurately simulated under harsh environment.

## Figures and Tables

**Table 1:** Deflection angle in models with different pore–crack sizes and distances.

	D/r	2	3	4	5	6
r						
0.4		*1	*4	*7	*10	*13
		25°	45°	55°	65°	75°
0.8		*2	*5	*8	*11	*14
		30°	50°	60°	70°	75°
1.2		*3	*6	*9	*12	*15
		35°	55°	65°	70°	80°

## Data Availability

Data are contained within the article.
